# Isolated Starvation Ketoacidosis: A Rare Cause of Severe Metabolic Acidosis Presenting with a pH Less than 7

**DOI:** 10.7759/cureus.4086

**Published:** 2019-02-17

**Authors:** Ateeq Mubarik, Aamani Jupalli, Arshad Muhammad Iqbal, Salman Muddassir, Abdulmagid Eddib

**Affiliations:** 1 Internal Medicine, Oak Hill Hospital, Brooksville, USA

**Keywords:** starvation, ketoacidosis, metabolic acidosis, anion gap metabolic acidosis, ph, ketosis, isk, agma

## Abstract

Anion gap metabolic acidosis (AGMA) occurs when an anion gap exists along with metabolic acidosis, most commonly due to diabetic ketoacidosis (DKA) and lactic acidosis (LA). Isolated starvation ketoacidosis (ISK) is one of the rare causes of AGMA; however, it usually presents with a mild disturbance in pH. We report a rare case of a 45-year-old female with previously diagnosed squamous cell cancer (SCC) of the larynx. She presented to the emergency department complaining of difficulty in breathing following laryngectomy and tracheostomy for SCC. Her laboratory results on admission were consistent for isolated starvation ketoacidosis and the patient responded quickly to the appropriate treatment.

## Introduction

Metabolic acidosis is characterized by a rise in acid production or decrease in acid elimination by the kidneys, usually triggering metabolic abnormalities. The normal blood pH is between 7.38 and 7.42. Lactic acidosis (LA) and diabetic ketoacidosis (DKA) are among the top leading causes for metabolic acidosis in routine critical care practice [1]. Ketoacidosis is usually caused by diabetes mellitus (DM) Type 1 or Type 2 ketone prone, alcohol or starvation [2]. Isolated starvation ketoacidosis (ISK) is a rare but well-understood phenomenon encountered by physicians in routine practice. In the non-pregnant population, several case reports of mild ISK have been documented [3,4]. There are no case reports documenting severe starvation ketoacidosis with a pH less than 7 in a non-pregnant population.

## Case presentation

A 45-year-old woman with a substantial past medical history of squamous cell cancer (SCC) was treated with laryngectomy and offered tracheostomy. She presented in the emergency department with complaints of shortness of breath (SOB). Her shortness of breath was getting progressively worse starting two days prior to admission. She denied any fevers, chills, sick contacts, nausea, abdominal pain, or diarrhea. She specified that she had actually been drinking a lot more water than regular prior to admission.

On admission, her vital signs revealed a blood pressure of 101/73 mmHg, a heart rate of 91 beats/min, a temperature of 37.4 degree Celsius, and a respiratory rate of 26 breaths/min. Her body mass index (BMI) was 18.6 kg/m2. Physical examination revealed a sick-appearing woman in severe respiratory distress using accessory muscles. She had a dry mucous membrane with poor skin turgor. The rest of the physical examination was unremarkable.

On laboratory assessment, the hemoglobin was 11.9 mg/dl, leukocyte count 3.6/mm3, serum creatinine 0.8 mg/dl, potassium 3.3 mmol/L, chloride 110 mmol/L, sodium 148 mmol/L, and bicarbonate 6 mmol/L. She had high anion gap metabolic acidosis (AGMA), (anion gap (AG) = 22). Her serum albumin on admission was 4.2 g/L, urine analysis revealed 80 mg/dl ketones, and serum lactate was 1.9 mmol/L. Furthermore, her liver enzymes revealed aspartate aminotransferase (AST) = 48 units/l, alanine aminotransferase (ALT) = 82 units/l, and alkaline phosphatase 199 units/l. Additionally, her blood glucose level was 133 mg/dl, salicylates = 6.8 mg/dl, and acetaminophen level was <2.0 ug/ml. Her blood alcohol level was normal and chest X-ray (CXR) on admission did not show any sign of acute cardiopulmonary problems.

Based on the initial evaluation, she received stoma suctioning and was placed on high O2 via a tracheostomy mask. Her history of laryngeal cancer, mild tachycardia and hypoxia raised the suspicion of pulmonary embolism, so a chest computed tomography angiography (CTA) was ordered. The chest CTA was negative for pulmonary embolism but showed mild emphysema.

In addition to that, an arterial blood gas (ABG) test was done, which revealed a high anion gap metabolic acidosis (HAGMA) as presented in Table 1. The patient continued to hyperventilate to compensate for the acidosis and was subsequently intubated.

**Table 1:** Laboratory values upon admission and during intensive care unit course

Laboratory Value	Day 1 (05:08)	Day 1 (15:05)	Day 2 (03:19)
Serum pH	6.88	7.236	7.457
pCO2 (mmHg)	21.0	40.5	30.6
Serum HCO3 (mmol/L)	6	19	26
Anion gap (mmol/L)	32	14	10
Delta anion gap/ Delta HCO3	1.11	0.4	1.0
Serum lactate (mmol/L)	1.9	-	-
Urine ketone (mg/dL)	80	38	6
Serum osmolality (mOsm/kg)	276	-	-
Serum potassium (mmol/L)	3.3	3.3	3.0
Serum phosphorus (mmol/L)	4.2	-	0.6
Serum hemoglobin (g/dL)	11.9	-	10.7
Serum creatinine (g/dL)	0.9	1.2	1.2

Differential diagnoses including carbon monoxide poisoning, aminoglycoside toxicity, methanol, uremia, diabetic ketoacidosis (DKA), alcoholic ketosis, acetaminophen toxicity, iron ingestion, lactic acidosis, ethanol toxicity, salicylate toxicity, and aspirin ingestion were investigated. However, given the patient's normal acetaminophen, salicylate, lactic acid, and ethanol levels, these etiologies were ruled out. Furthermore, serum osmolality was normal and the osmolal gap was less than 10. Our differential diagnosis was further narrowed due to the presence of elevated ketones. Given the patient's malnourished state, the most likely cause of HAGMA in this patient was starvation ketosis. The patient was started on 5% dextrose water and sodium bicarbonate drip, and tube feeds. The ABG level swiftly improved within three days and she was extubated.

After four days of intensive care unit (ICU) course, she was downgraded and later on discharged after detailed counseling from a nutritionist regarding a balanced diet and avoiding fasting. She was followed up after three weeks of discharge with significant improvement on repeat basic metabolic panel with bicarbonate of 25 mmol/L, sodium 138 mmol/L, potassium 3.8 mmol/L, and chloride 102 mmol/L.

## Discussion

Acetone, acetoacetate, as well as beta-hydroxybutyrate are ketone bodies that accumulate in the blood as a result of the imbalance between production and elimination. The liver, brain, muscle, as well as kidney, can all consume ketone bodies; however, the liver is the only organ which contributes significantly to the overall accumulation of ketone bodies in the blood [5,6]. Furthermore, the liver is the key organ in the production of ketone bodies under the influence of hormones like insulin and glucagon. Also, the rate of hepatic ketone manufacturing depends on various factors including, but not restricted to, substrate accessibility, regulative hormones (insulin and glucagon), and concentration of end products while the rate of elimination of ketone bodies from the blood depends on the consumption by peripheral tissues, specifically skeletal muscles [7].

Additionally, ketone body production maximizes after starvation for at least three days [8]. Interestingly, in healthy individuals, starvation exceeding fourteen days causes significant ketoacidosis but the pH remains above 7.30 [9]. During pregnancy, especially in the third-trimester, ketone production starts after 12 hours of starvation [10]. In the pregnant population, response to starvation is in the form of increased ketone production [11]. Frise et al. in his case series reported four cases of starvation ketoacidosis associated with pregnancy with the lowest pH of 7.27 [12]. Lakkis et al. reported a case of severe metabolic acidosis with a pH of 7.09 due to starvation in a patient with spinal muscle atrophy due to decreased oxidation of ketone bodies by muscle fibers [13].

Likewise, alcohol can also induce ketoacidosis, particularly in individuals experiencing extreme as well as persistent alcoholic dependence irrespective of the age [14]. Binge alcohol drinkers are a lot more prone to ketosis because of decreased calorie consumption as well as reduced glycogen storage [15]. In our patient, the contribution of chronic alcohol abuse cannot be excluded as the possible cause for severe metabolic ketoacidosis although the blood ethanol levels were normal and the patient denied any recent alcohol use. Data suggests that alcohol level can be normal in patients with alcoholic ketoacidosis [16]. The level of free fatty acids (FFA) can be used to differentiate between starvation ketoacidosis and alcoholic ketoacidosis where the level of FFA is higher in alcoholic ketoacidosis as compared to starvation ketoacidosis [17]. Our patient's FFA levels were mildly elevated.

The monitoring of AGMA is of vital relevance, and intravenous (IV) dextrose water is the standard therapy for standard ketoacidosis. Starvation ketoacidosis is treated with three varied strengths of dextrose water—5%, 10%, 50% [3]—and our patient responded enormously to 5% dextrose water (Table 1) [4]. Additionally, we started tube feeding the patient, which helped to further stimulate insulin and inhibit the formation of ketone bodies. In order to prevent Wernicke’s encephalopathy, we also injected thiamine prior to the start of IV dextrose water.

## Conclusions

This is a unique case as starvation ketoacidosis usually does not present with a pH less than 7. This was as a result of our patient starving for more than two weeks prior to admission. Our patient was treated to compensate appropriately for severe metabolic ketoacidosis and subsequently intubated. Based on the experience with our patient, we strongly believe that a patient can exhibit deadly, severe metabolic ketoacidosis with a pH less than 7 due to starvation. After ruling out other usual sources of metabolic acidosis and given the quick restoration of acidosis and ketosis with intravenous dextrose water and a small amount of sodium bicarbonate, starvation was deemed to be the most likely source of our patient’s metabolic acidosis.
